# Bovine neonatal pancytopenia - Comparative proteomic characterization of two BVD vaccines and the producer cell surface proteome (MDBK)

**DOI:** 10.1186/1746-6148-9-18

**Published:** 2013-01-23

**Authors:** Kerstin N Euler, Stefanie M Hauck, Marius Ueffing, Cornelia A Deeg

**Affiliations:** 1 Institute of Animal Physiology, Department of Veterinary Sciences, LMU Munich, Veterinärstr. 13, München D-80539, Germany; 2 Research Unit for Protein Science, Helmholtz Zentrum München - Germany Research Center for Environmental Health (GmbH), Ingolstädter Landstr. 1, Neuherberg, D-85764, Germany; 3 Centre of Ophthalmology, Institute for Ophthalmic Research, University of Tübingen, Röntgenweg 11, Tübingen, D-72076, Germany

## Abstract

**Background:**

Bovine neonatal pancytopenia (BNP) is a disease syndrome in newborn calves of up to four weeks of age, first observed in southern Germany in 2006. By now, cases have been reported in several countries around the globe. Many affected calves die within days due to multiple haemorrhages, thrombocytopenia, leukocytopenia and bone marrow depletion. A certain vaccine directed against Bovine Virus Diarrhoea Virus (BVDV) was recently shown to be associated with BNP pathogenesis. Immunized cows develop alloantibodies that are transferred to newborn calves via colostrum intake. In order to further elucidate BNP pathogenesis, the purpose of this study was to characterize and compare the protein composition of the associated vaccine to another vaccine directed against BVDV not related to BNP and the cell surface proteome of MDBK (Madin-Darby Bovine Kidney) cells, the cell line used for production of the associated vaccine.

**Results:**

By SDS-PAGE and mass spectrometry, we were able to detect several coagulation-related and immune modulatory proteins, as well as cellular and serum derived molecules being shared between the associated vaccine and MDBK cells. Furthermore, the number of proteins identified in the BNP related vaccine was almost as high as the number of surface proteins detected on MDBK cells and exceeded the amount of proteins identified in the non-BNP related vaccine over 3.5 fold. The great amount of shared cellular and serum derived proteins confirm that the BNP associated vaccine contained many molecules originating from MDBK cells and vaccine production.

**Conclusions:**

The respective vaccine was not purified enough to prevent the development of alloantibodies. To narrow down possible candidate proteins, those most likely to represent a trigger for BNP pathogenesis are presented in this study, giving a fundament for further analysis in future research.

## Background

Bovine neonatal pancytopenia (BNP) is a disease transferred by colostral alloantibodies binding to peripheral blood-derived leukocytes and platelet antigens of calves [1]. Remarkably, calves develop a severe thrombocytopenia and leukocytopenia within few hours after passive transfer of colostral antibodies to blood and die within several days from bleeding disorder and bone marrow depletion [1,2]. Respective alloantibodies responsible for BNP can develop in cows previously vaccinated with a specific Bovine Viral Diarrhoea (BVD) vaccine (PregSure BVD; Pfizer, Berlin, Germany; vaccine A) [1]. Colostra of these cows transfer BNP to healthy calves, indicating a commonly expressed target antigen in responding calves [2]. Alloantibodies are also detectable in blood of respective BNP dams [1], suggesting their development to be systemically and not directly in udder. Further immunological characterization of these antibodies revealed that they were of IgG1 subclass [3]. IgG1 antibodies reflect a Th2-response in cows. So far, Major histocompatibility complex class I (MHC I) was identified as one potential BNP alloantigen in two independent studies, one demonstrating alloimmune reactions to MDBK cell lysates of BNP donors [4] and the other describing responses to vaccine A derived proteins [5], but there are also other data indicating a different alloantigen [3]. Defects in coagulation of platelets, the decline of platelets and thus the low platelet count are the main cause for the multiple haemorrhages leading to the death of affected calves. However, the antigen(s) appear(s) to be also expressed on mature PBL as Assad et al. demonstrated binding of colostrum-derived antibodies to several PBL subsets and platelets [3], and further on hematopoietic progenitor cells of the myeloid and lymphoid lineages in the bone marrow, as these are, according to Laming et al., comprised already 24 hours after colostrum intake and show a drastic decline within the first 6 days after colostrum intake [6].

BNP dams clearly develop alloreactive responses after vaccination with vaccine A, but not with other BVD vaccines [7,8]. Immunization of experimental calves and guinea pigs with this vaccine led to generation of alloantibodies [9], which were also cross-reactive with MDBK cells [10], the cell line used for production of vaccine A [9]. MDBK line was derived from the renal tissue of an adult steer in 1957 [10]. MDBK cells are susceptible to infection with BVDV and a number of other viruses, including vesicular stomatitis virus, infectious bovine rhinotracheitis virus, bovine parvovirus, bovine adenovirus I and III, and parainfluenza virus 3 [11]. MDBK cells exhibit resistance to poliovirus 2 and are negative for reverse transcriptase.

Therefore, the goal of this study was to characterize and compare protein expression of MDBK cells, BNP associated vaccine A, and - as negative control to contrast the BVD virus associated constituents - a live-attenuated BVD vaccine unrelated to BNP (Vacoviron FS; Merial, Hallbergmoos, Germany; vaccine B) by label free mass spectrometry. The results may improve our understanding of differences in vaccine composition that could account for alloantibody formation in BNP dams.

## Results

### BNP associated BVD vaccine contains a variety of different proteins

**Figure 1:**
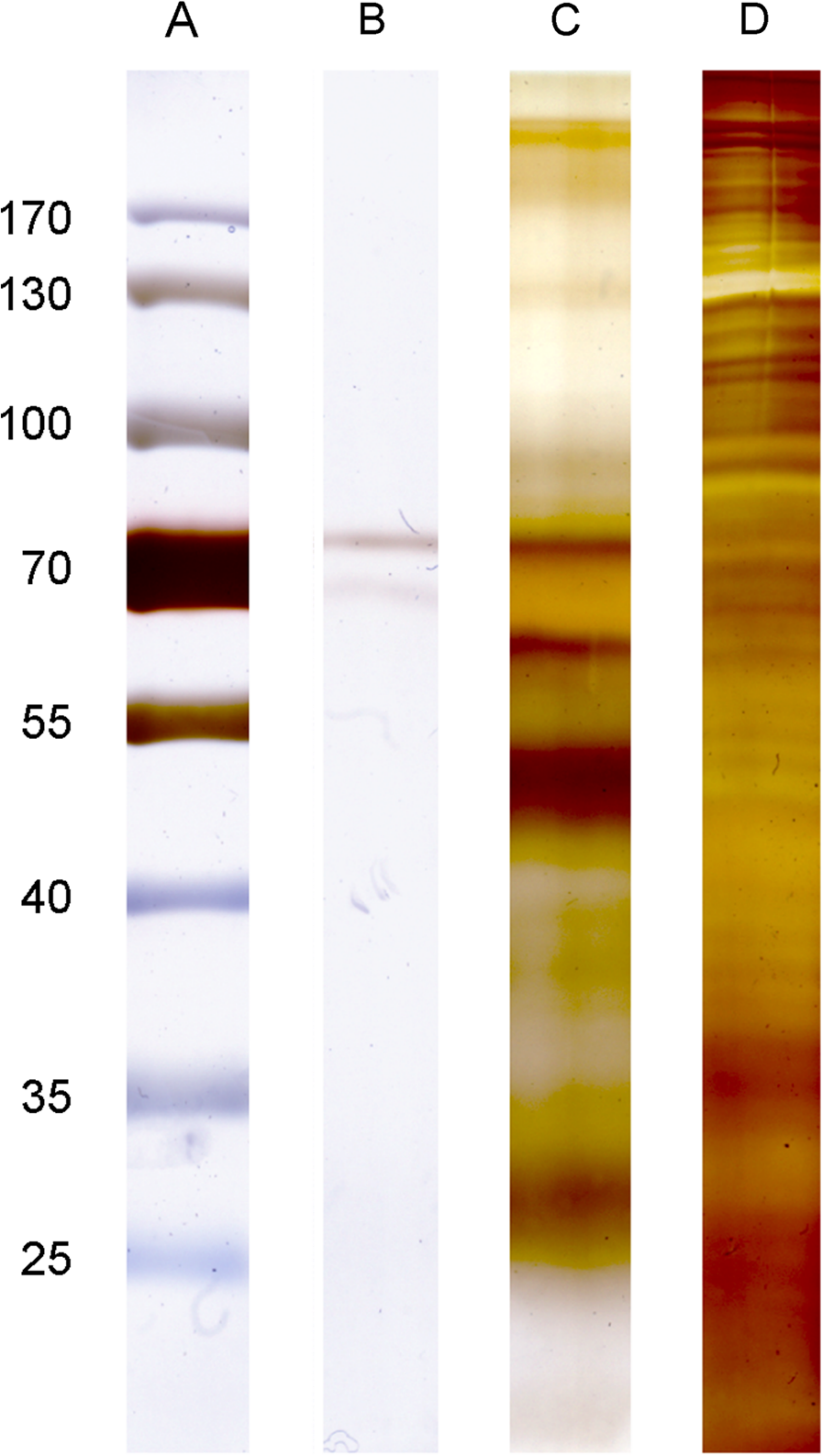
**1D gel demonstrating diverging protein concentration in BVD vaccines and MDBK cell lysate, silver stained.** 50 μg of PregSure BVD (vaccine B), the according volume Vacoviron FS (vaccine A) and 50 μg MDBK cell lysate were loaded on a 10% SDS-Gel, resolved by SDS-PAGE and silver stained. A: Molecular weight marker. B: Vaccine B. C: Vaccine A. D: MDBK cell lysate

First evaluation of protein composition of different BVD vaccines showed a clear difference between vaccine B (Figure 1B) and A (Figure 1C). Vaccine A, related to BNP, included many different proteins in comparison to vaccine B which is not associated with BNP. Quantitative staining with colloidal Coomassie demonstrated a remarkable difference in protein concentration of vaccine A compared to vaccine B. In addition, silver staining confirmed little protein content in vaccine B (Figure 1B) whereas vaccine A (Figure 1C) resembled the composition of MDBK cell proteome (Figure 1D).

We therefore decided to identify all proteins of respective samples with LC-MS/MS to categorize duplicate proteins in vaccine A and MDBK cells.

### Mass spectrometric classification of protein content confirms large overlap between MDBK and PregSure BVD proteomes

A total of 310 proteins were identified by LC-MS/MS in this study (see Additional file 1). 43 proteins were identified with two or more peptides in vaccine B, a live-attenuated vaccine which is not related to BNP. In contrast, 159 proteins were detectable in vaccine A (vaccination with this vaccine strongly correlates with BNP occurrence) and 163 proteins on MDBK cell surface of which 66% are allocated to the Gene ontology (GO) project terms “plasma membrane”, “cell surface” or “extracellular”. There was an overlap of eight proteins in all three samples analyzed. Eleven proteins were present only in both vaccines A and B, but not on MDBK cells. Three other proteins, alpha-S1-casein, kappa-casein and keratin-5 appeared concurrently in vaccine B and MDBK cells. Two BVD virus polypro-tein peptides were detected in both vaccines (vaccine A: EMBL Database Accession Number: ABP57735.1; EMBL Database (http://www.ebi.ac.uk/embl/; vaccine B: EMBL Database Accession Number AAC61755.1).

Between vaccine A and MDBK cells, we detected 25 shared proteins (Tables 1 and 2). Among these were 17 proteins representing cellular components (Table 1, proteins 1–12, 14, 15, 17, 18 and 25), e.g. histone H2B type 1 and mitochondrial proteins ATP synthase subunits alpha, beta and adenylate kinase 2 (AK 2). Further, we detected ribosomal proteins S3, S4 and L7a and representatives of cytoplasmatic protein fraction. The latter were structural proteins of the cytoskeleton, such as actin, or proteins in charge of energy balance like glyceraldehyde-3-phosphate dehydrogenase (GAPDH). Also, the chaperone heat shock cognate 70 kDa protein 8 (hsc70) and an inhibitor of cysteine and serine proteases, endopin-2 [12] belong to this fraction. Besides, two membrane-bound proteins, namely sodium/potassium-transporting ATPase subunit alpha-1, essential for osmoregulation [13], and MHC I heavy chain isoform 1, which is expressed on all mammalian nucleated cells [14], were identified (Table 1, proteins 12 and 14).

**Table 1:** Shared proteins between vaccine A and MDBK cell surface

**No.**	**Identified Protein**	**Accession Number**	**MW (kDa)**
1	Histone H2B type 1	ENSBTAP00000024155	25
2	40S ribosomal protein S3	ENSBTAP00000003962	27
3	40S ribosomal protein S4	ENSBTAP00000052226	30
4	60S ribosomal protein L7a	ENSBTAP00000015358	30
5	Actin, cytoplasmic 2, N-terminally processed	ENSBTAP00000008132	42
6	Alpha-actinin-4	ENSBTAP00000014894	105
7	Keratin 8	ENSBTAP00000001108	54
8	Glyceraldehyde-3-phosphate dehydrogenase	ENSBTAP00000037577	36
9	Alpha-enolase	ENSBTAP00000017839	47
10	Pyruvate kinase	ENSBTAP00000044619	61
11	Endopin-2	ENSBTAP00000011576	47
12	Sodium/potassium-transporting ATPase subunit alpha-1	ENSBTAP00000001646	113
13	Alpha-2-HS-glycoprotein	ENSBTAP00000000673	38
14	MHC class I heavy chain isoform 1	ENSBTAP00000031126	40
15	Heat shock cognate 70 kDa protein 8	ENSBTAP00000017497	71
16	Hemopexin	ENSBTAP00000004635	52
17	ATP synthase subunit alpha, mitochondrial	ENSBTAP00000003259	60
18	ATP synthase subunit beta, mitochondrial	ENSBTAP00000017710	56
19	Complement C3	ENSBTAP00000022979	187
20	Complement C4	ENSBTAP00000009019	102
21	Vitamin D-binding protein	ENSBTAP00000033386	53
22	Thrombospondin-1	ENSBTAP00000002600	129
23	Alpha-1-acid-glycoprotein	ENSBTAP00000022991	23
24	Alpha-1-antiproteinase	ENSBTAP00000004927	46
25	Adenylate kinase 2, mitochondrial	ENSBTAP00000023406	26

25 proteins could be co-identified in vaccine A and on MDBK cells. Proteins listed were identified by LC-MS/MS with a probability score that is significant with p < 0.05 if the confidence score was >30 at a significance threshold for the Mascot result of p ≤ 0.01. Accession number as listed on Ensembl database (http://www.ensembl.org).

**Table 2:** Abundance of proteins in vaccine A shared with MDBK

**No.**	**Identified Protein**	**Accession Number**	**MW (kDa)**	**total spectra (%)**	**unique peptide count**	**total spectra count**	**Coverage (%)**
1	Complement C4	ENSBTAP00000009019	102	0.150	36	81	30.0
2	Actin, cytoplasmic 2, N-terminally processed	ENSBTAP00000008132	42	0.120	10	64	45.0
3	Pyruvate kinase	ENSBTAP00000044619	61	0.066	17	35	40.0
4	keratin, type II cytoskeletal 8	ENSBTAP00000001108	54	0.057	13	30	31.0
5	Histone H2B type 1	ENSBTAP00000024155	25	0.055	4	29	35.0
6	Thrombospondin-1	ENSBTAP00000002600	129	0.043	15	23	17.0
7	Glyceraldehyde-3-phosphate dehydrogenase	ENSBTAP00000037577	36	0.038	8	20	37.0
8	Alpha-1-antiproteinase	ENSBTAP00000004927	46	0.032	2	17	7.2
9	Heat shock cognate 70 kDa protein 8	ENSBTAP00000017497	71	0.032	12	17	29.0
10	ATP synthase subunit beta, mitochondrial	ENSBTAP00000017710	56	0.028	9	15	25.0
11	Alpha-actinin-4	ENSBTAP00000014894	105	0.026	11	14	19.0
12	Alpha-1-acid glycoprotein	ENSBTAP00000022991	23	0.021	5	11	31.0
13	Vitamin D-binding protein	ENSBTAP00000033386	53	0.019	2	10	7.0
14	Adenylate kinase 2, mitochondrial	ENSBTAP00000023406	26	0.017	7	9	42.0
15	40S ribosomal protein S3	ENSBTAP00000003962	27	0.013	3	7	15.0
16	Alpha-enolase	ENSBTAP00000017839	47	0.013	5	7	18.0
17	ATP synthase subunit alpha, mitochondrial	ENSBTAP00000010806	60	0.011	4	6	12.0
18	Endopin 2	ENSBTAP00000011576	47	0.009	3	5	13.0
19	Hemopexin	ENSBTAP00000004635	52	0.009	3	5	12.0
20	Alpha-2-HS-glycoprotein	ENSBTAP00000000673	38	0.008	3	4	17.0
21	Na^+^/K^+^-transporting ATPase subunit alpha-1	ENSBTAP00000001646	113	0.008	3	4	3.3
22	40S ribosomal protein S4	ENSBTAP00000052226	30	0.006	3	3	12.0
23	Complement C3	ENSBTAP00000022979	187	0.006	3	3	4.9
24	60S ribosomal protein L7a	ENSBTAP00000015358	30	0.004	2	2	13.0
25	MHC class I heavy chain isoform 1	ENSBTAP00000031126	40	0.004	2	2	6.4

Proteins identified concurrently in vaccine A and on MDBK cell surface by LC-MS/MS are shown in the order of the protein percentage of total spectra, reflecting their abundance in vaccine A, from lowest to highest concentration. Accession number as listed on Ensembl database (http://www.ensembl.org). Proteins listed were identified with a probability score that is significant with p < 0.05 if the confidence score was >30 at a significance threshold for the Mascot result of p ≤ 0.01. *total spectra (%):* percentage of total spectra assigned to respective protein. *unique peptide count:* number of unique peptides for the identified protein*. total spectra:* number of total spectra assigned to the identified protein. *coverage (%):* percentage of total amino acid sequence covered with the identified peptides.

Further, eight serum-derived proteins were detected (Table 1, proteins 13, 16 and 19–24). Complement 3 (C3) and C4 are abundant blood components and members of complement cascade [15]. Alpha-2-HS-glycoprotein, vitamin D-binding protein (VDBP), alpha-1-acid-glycoprotein, and hemopexin represent important transport proteins [16-19], alpha-1-antiproteinase participates in wound-healing [20] and thrombospondin-1 (TSP-1) is associated with platelet aggregation [21]. The protein percentage of total spectra, the number of unique peptides of the proteins identified, the number of total spectra identified for respective proteins and the percentage of amino acids identified in vaccine A are presented in Table 2. According to these parameters, the most abundant of the proteins detected concurrently in vaccine A and on MDBK cell surface was C4 (Table 2, protein 1), while 60S ribosomal protein L7a and MHC class I heavy chain (Table 2, proteins 24 and 25) were the least abundant.

## Discussion

For the first time, we characterized the proteome of two BVD vaccines and compared their protein content and concentration in regard to BNP pathogenesis. The inactivated BVD vaccine A contained over 3.5 fold more proteins than the live-attenuated vaccine B and still over twice as many compared to a meningococcal vaccine*,* where 47 proteins were identified by similar proteomic approaches [22]. Total protein quantity of vaccine A and MDBK cells was almost equal, underlining our findings that the protein content of vaccine A and the host cell line surface resemble each other (Figure 1C and D). This indicates that the vaccine antigens have not or only insufficiently been purified. Further, the number of individual proteins was much lower in vaccine B compared to vaccine A, which was expected of the live-attenuated vaccine B and the reason why it was used as a qualitative negative control. Also, the protein concentrations of both vaccines differed greatly. Thus, the administered amount of protein in vaccine A exceeds the one applied when immunizing with vaccine B according to dosage instructions.

Furthermore, we provided proteomic characterization of MDBK cell surface. 66% of identified proteins on MDBK cells were attributed with the Gene Ontology project terms “plasma membrane”, “cell surface” or “extracellular”, indicating a high enrichment of proteins already known to be allocated to the plasma membrane. Although this certainly reflects technical limitations that are inherent in most membrane-associated fraction enrichment methods, as one third of these identified proteins are currently not attributed as cell surface proteins, it could on the other hand also point out that several proteins might be additionally expressed on the cell surface, but that their presence on this location has not yet been described. One prominent example is ATP Synthase, an ubiquitous protein of the oxidative phosphorylation pathway which was considered to be an exclusively mitochondrial protein for decades, but was discovered recently on the cell surface of a great variety of cell types [23]. MDBK cells are often used as culture system for growing attenuated viruses in vaccine production [24], e.g. BVDV [9]. Proteins detected in all preparations or overlapping in one vaccine and MDBK cells indicate an impurity of the vaccines with homologous proteins due to manufacturing processes. Proteins present in both vaccines suggest a vaccine specific role, possibly as adjuvants. Calreticulin, for instance, which was detected in both vaccines, aids in antigen presentation promoting antigen contact to MHC I [25]. This immune enhancing property constitutes its use as compound of vaccines, especially tumor vaccines [25].

By characterizing the host cell line used in manufacturing vaccine A, vaccine A itself and the qualitative negative control vaccine B proteomically, we were able to narrow down proteins administered through vaccine A to several interesting candidates which might be involved in pathogenesis of BNP. Here we discuss proteins that we believe deserve further evaluation because they offer interesting properties such as an association with clotting, being previously reported to be involved in immune diseases that show symptoms similar to the ones seen in BNP or a location on cell types affected in BNP pathogenesis. The rapid decrease in the number of platelets and leukocytes in the circulating blood within the first few hours after colostrum intake is most likely directly antibody-mediated and probably not due to bone marrow depletion. Whereas the haematopoietic progenitor cells are compromised in their ability to form colonies as early as 24 hours after uptake of colostral antibodies, the decrease of haematopoietic progenitor cells develops over a longer period of time [6]. Thus, we suspect the antigen or antigens to be located on PBL, platelets and, in regard to the bone marrow depletion, also on haematopoietic progenitor cells of the myeloid and lymphoid lineages.

Interestingly, Pfizer used a new potent adjuvant in vaccine A, containing immunostimulating complexes. These structures envelop the antigen enhancing its uptake into immune-modulatory cells [26]. The saponin mixture Quil A is part of these complexes. Its use in human vaccines was restricted based on its high toxicity and haemolytic effect [27], until introduction of respective complexes reduced these effects [28]. Further, the adjuvant also contains drakeol 5, a regular mineral oil, which is often used in veterinary vaccine production and helps stabilize the emulsion of vaccine components. Since it cannot be metabolized, it maintains a slow release of antigen and remains in the injected tissue as an oil pool or depot. It is also known to lead to a prolonged irritation at the injection site, thus reflecting the possible side effects of the vaccine according to the instruction leaflet [29].

The adjuvant is believed to have potentiated reactions to alloantigens present in vaccine A [9]. To avoid a false negative selection of our candidate proteins, we chose a live-attenuated vaccine as negative control. On the one hand, these vaccines generally contain less protein than inactivated vaccines. Acquiring another BVD vaccine with a high protein variety, as other inactivated vaccines predictably would show, would have beared the risk of ruling out valuable candidate proteins that might only lead to antibody production due to the effect of the new adjuvant used in vaccine A. On the other hand, a live-attenuated vaccine more likely provides proteins that are only present due to certain technical limitations of manufacturing processes in vaccine production. 

Pfizer withdrew the registration of vaccine A in Europe in June 2010 (in Germany even two months earlier, in April 2010). It took another 14 months until vaccine A was drawn from the market in New Zealand in August 2011, right after the first case was reported there. It is worth mentioning that there is a close correlation of the time period when vaccine A was brought to market in Europe in 2004, with cases of haemorrhagic diatheses occurring cumulatively in 2007, and it being available for vaccination in New Zealand in May 2008 where also, three years later, the first diagnosed case of BNP occurred in August 2011.

Analysing the MDBK cell surface proteome (see Additional file 1), we detected several interesting BNP alloantigen candidates, such as integrin alpha-IIB (CD41) and von Willebrand factor, that have essential functions in clotting [30,31]. Although they did not appear in vaccine A, we hypothesized that, since the oily formulation of the vaccine made it difficult to handle it technically [5], the antigen might have been undetectable by LC-MS/MS. Thus, we tried to validate CD41 in vaccine A by Western blot analysis with an antibody directed against human CD41 protein (data not shown) since this was the only available commercial antibody at this point of time, but did not succeed in verifying our hypothesis.

Proteins identified in vaccine A and MDBK cells (Tables 1 and 2) demonstrate a clear difference in composition of vaccine A and B. This group most likely contains candidate proteins capable of triggering production of alloantibodies in BNP pathogenesis.

Several of these proteins are common cellular components (Table 1, proteins 1–12, 14, 15, 17, 18 and 25). For instance, GAPDH contributes to cellular energy supply and is considered a housekeeping protein [32]. Occurrence of these MDBK cell associated proteins in vaccine A (and lack of these in vaccine B) confirm high amount of cellular debris in vaccine A.

Previous studies described vaccine A being contaminated with MHC I, which was also present on MDBK cells and supposedly induced alloreactive antibodies in BNP dams [4,5]. Although we detected MHC I in both preparations (Table 1, protein 14 and Table 2, protein 25), we do not support the hypothesis that MHC I alloantibodies cause BNP pathogenesis. We could show by immunological characterization that BNP alloantibodies have another binding pattern than MHC I expression [3]. There was a difference in alloantibody binding to different lymphocyte subsets whereas MHC I is evenly expressed on all lymphocytes [14]. Also, MHC I was the least abundant protein according to the protein percentage of spectral counts in our LC-MS/MS results (Table 2, protein 25). Further, human MHC I (HLA I) alloantibodies contribute to transfusion-related acute lung injury (TRALI), a frequent complication in transfusion medicine [33]. Affected patients develop acute lung edema within six hours of transfusion, caused by administration of a great amount of antibodies from donor blood directly into the recipient’s circulation. This contrasts to BNP, where calves suffer fatal bleeding up to four weeks after colostrum intake. Although MHC I antibodies were the most frequent alloantibodies (73%) detected in TRALI, they were only weak triggers and did not cause any fatal case [34]. Still, not all important transfusion alloantigens are detected for humans. This was recently shown by Greinacher et al. who identified a biallelic polymorphism in human neutrophile antigen 3a (HNA-3a) as alloantigen in TRALI, resulting from a single-nucleotide exchange in the choline transporter-like protein [33]. HNA-3a is a more potent alloantigen causing a more severe and fatal course of TRALI. Identification of this alloantigen was technically challenging and covered by prominent anti MHC I immune reaction of respective patients. This could also be the case in BNP, therefore investigation of involved alloantigens continues until pathogenesis induction is proven by a monospecific antibody.

Interestingly, we identified several proteins that were previously described as targets in auto- or alloimmune-mediated diseases. Alpha-enolase (Table 1, protein 9 and Table 2, protein 16), a glycolytic enzyme and plasminogen-binding protein on leukocytes [35], is an autoantigen in rheumatoid arthritis [36] and Behcet’s disease [37]. Hsc70 (Table 1, protein 15 and Table 2, protein 9) acts as chaperone of proteins to other chaperones or membranes and takes part in cellular thermo tolerance [38]. Antibodies against heat shock proteins were detectable in many autoimmune diseases, e.g. myasthenia gravis [39]. ATP synthase (Table 1, proteins 17 and 18 and Table 2, proteins 10 and 17) catalyzes ATP from ADP at the mitochondrial membrane and is present on surface of various cells, including endothelial cells, where it is an autoantigen in immune-mediated vasculitides [40]. Although C3 and C4 (Table 1, proteins 19 and 20 and Table 2, proteins 1 and 23) play a significant role in the innate immune system [15] a mouse model demonstrated that deficiency in C3 can be compensated by thrombin [41] and a genetically determined deficiency of C4 increases the risk for developing systemic lupus erythematosus [42]. Alloantibodies directed against this group of proteins, however, seem unlikely to be involved in BNP pathogenesis as their functions and the immune-mediated diseases do not correlate with bleeding disorders.

In addition, we were able to detect candidate proteins directly affecting cell types reported as BNP targets, namely platelets, monocytes, granulocytes, B- and T-cells [3].

Vitamin D binding protein (Table 1, protein 21 and Table 2, protein 13) acts as transport protein for vitamin D in plasma, and is present on human neutrophils, B-cells and a subset of T-cells, enhancing C5a chemotaxis of macrophages and neutrophils [43]. Alloantibodies against these cells could explain several symptoms of BNP, e.g. lymphocytopenia and neutropenia [1].

A further interesting BNP alloantigen candidate was thrombospondin-1 (TSP-1; Table 1, protein 22 and Table 2, protein 6), a protein secreted by and bound on surface of various cells including endothelial cells, monocytes, macrophages, and platelets [44]. It is an important interactor of coagulation proteins such as fibrinogen, fibrin, integrins and neutrophil elastase. It activates leukocytes, inhibits T-cell effectors and directly induces T-cell apoptosis [44]. Further, it promotes T-cell adhesion and chemotaxis [44]. TSP-1 null mice showed increased bleeding and colonic inflammation in acute induced colitis compared to controls [44]. Therefore, TSP-1 merits further exploration as possible BNP alloantigen.

Alpha-1-acid-glycoprotein (Table 1, protein 23 and Table 2, protein 12) was detected, a plasma protein with many immune-modulatory functions, e.g. expression of anti-inflammatory cytokines by macrophages, decreasing chemotaxis of neutrophils and inhibiting proliferation of lymphocytes [19]. It is also synthesized during granulocyte differentiation and stored in granules, but can further be produced at inflammation sites, e.g. by endothelial cells and activated macrophages, or transported to affected tissues by defensive cells, for instance neutrophils [19]. This protein has several functions in BNP pathogenesis associated pathways, therefore, alloantibodies against alpha-1-acid-glycoprotein might potentially play an important role in development of BNP.

An additional interesting candidate was alpha-1-antiproteinase (Table 1, protein 24 and Table 2, protein 8). It plays an important role in wound-healing [20], inhibiting plasmin and activating plasminogen and thrombin, and also inhibits haematopoietic stem cell mobilization in bone marrow [45]. Further, *in vitro* studies showed an increase of lipopolysaccharide-mediated macrophage activation and anti-inflammatory effects on B-cells, demonstrating an additional role in immune regulation [46]. Whereas inactivating mutations and deficiencies of alpha-1-antiproteinase only resulted in elastase-induced tissue damage, such as skin hyperextensibility [47], chronic obstructive pulmonary disease and liver disease [46] in humans, but not in bleeding disorders, the Pittsburgh mutation of alpha-1-antiproteinase results in a fatal haemorrhagic diathesis due to greatly enhanced protease-inhibitory effects on thrombin, thus blocking the coagulation cascade [46]. These functions of alpha-1-antiproteinase make it an interesting BNP alloantigen candidate. We will therefore further examine an involvement of anti-alpha-1-antiproteinase immune reactions in BNP pathogenesis.

Additionally, we identified adenylate kinase 2 (AK 2; Table 1, protein 25 and Table 2, protein 14) in vaccine A and MDBK cells. AK 2 is present in platelets, CD14^+^, CD19^+^, CD3^+^, CD56^+^, CD66b^+^ cells and in mononuclear cells obtained from bone marrow [48]. AK 2 mutations cause reticular dysgenesis, the most severe combined immunodeficiency in humans [48]. In reticular dysgenesis, mononuclear cells decrease in bone marrow, thus blocking myeloid differentiation at promyelotic stage [48]. It induces similar aberrations in the haemogram and bone marrow as described for BNP [2], resulting in monocytopenia, neutropenia, and lymphopenia combined with normal erythrocyte count and thrombocytopenia in some cases [49]. AK 2 knock-down in zebrafish caused similar alterations in leukocyte development, emphasizing the high level of conservation of AK 2 in different species [48]. Further, loss of AK 2 does neither interfere with development of immature hematopoietic cells nor erythropoiesis, but with lymphocyte development which in this case cannot be compensated by AK 1 due to lower or absent expression in respective cell types [48]. Affected cell subsets, the association to bone marrow and alterations in haemogram closely resemble alterations seen in BNP [2]. Thus, we consider AK 2 the most promising candidate in this study. However, further investigations have to aim at validating its role in BNP pathogenesis.

## Conclusions

We provide the first characterization of MDBK cell surface proteome and the proteomes of two BVD vaccines. BNP associated BVD vaccine A contained 159 proteins in comparison to BVD vaccine B (43 proteins, not BNP-related). The great overlap of cellular and serum-derived components confirmed contamination of the BNP related vaccine A with proteins from MDBK cell line used for vaccine production. Several interesting BNP candidate alloantigens could be detected within the overlap of proteins present in BNP related vaccine A and on MDBK cell surface. VDBP, TSP-1, alpha-1-acid-glycoprotein, alpha-1-antiproteinase, and AK 2 associate with coagulation and show a distribution resembling the affected tissues and cell populations in BNP. Alloantibodies against these proteins could play an important role in BNP pathogenesis and alloimmune reactions need to be further analyzed in future studies.

## Methods

### Sample preparation

MDBK cells (kindly provided by Falko Steinbach, Veterinary Laboratories Agency - Weybridge New Haw, Addlestone, Great Britain) were cultivated in RPMI-1640 medium supplemented with 10% heat-inactivated fetal calf serum (FCS) and 1% penicillin/streptomycin. Cells were maintained at 37°C and 5% CO_2._ For protein expression analysis by mass spectrometry, cells were harvested with Trypsin/EDTA (Biochrom, Berlin, Germany), washed twice with cold phosphate buffered saline, and centrifuged between washing steps at 4°C, 500 × g for 10 minutes (min). Prior to cell lysis, plasma membrane proteins of 2 × 10^6^ cells were labelled with 77.4 μg biotin. After 30 min rotation at 4°C and two centrifugation steps at 2800 × g and 16000 × g for 10 min each at 4°C, cells were lysed with 1% Nonidet P-40, 150 mM NaCl, 1× Roche Complete Protease Inhibitor, EDTA-free; 5 mM 2-Iodacetamide in 10 mM Tris–HCl pH 7.6 and biotinylated cell surface proteins were captured using Streptavidin-beads (IBA, Goettingen, Germany). After extensive washing to remove unspecifically bound proteins, captured proteins were cleaved through digesting beads overnight with trypsin (Promega, Mannheim, Germany) followed by incubation with glycerol-free PNGase F (New England Biolabs, Frankfurt/Main, Germany) at 37°C. Peptides were purified and concentrated using Pierce PepClean C18 spin columns (Thermo Fisher Scientific, Bonn, Germany) according to manufacturer’s protocol and subjected to analysis by liquid-chromatography mass spectrometry/mass spectrometry (LC-MS/MS).

### SDS-PAGE

After overnight precipitation of proteins from vaccine A with acetone at −20°C, the resulting pellet was resolubilized and both vaccines were resolved by 12% SDS-PAGE (sodium dodecyl sulfate polyacrylamide gel), followed by staining with Coomassie Brilliant Blue. After proteolysis with trypsin, samples were subjected to analysis by LC-MS/MS.

MDBK cells were solubilized with lysis buffer (9 M urea, 2 M thiourea, 1% DTT, 4% CHAPS, and 2.5 mM each of EGTA and EDTA) and protein content of both vaccines and the cell lysate was quantified with Bradford assay (Sigma-Aldrich, Deisenhofen, Germany).

Vaccine proteins and MDBK cell proteins were resolved by 10% SDS-PAGE, applying PageRuler Prestained Protein Ladder (Thermo Fisher) as reference marker. Gels were stained with silver and colloidal Coomassie [50] for a first comparison of protein content in vaccine A (related to BNP alloantibody generation [5]), vaccine B (unrelated to BNP alloantibody production) and MDBK cells.

### Mass spectrometry

LC-MS/MS analysis was performed as described before [51,52]. Briefly, peptides were separated on a reversed phase chromatography column (PepMap, 15 cm x 75 μm ID, 3 μm/100A pore size, LC Packings) operated on a nano-HPLC apparatus (Ultimate 3000, Dionex, Idstein, Germany). The nano-HPLC was connected to a linear quadrupole ion trap-Orbitrap (LTQ Orbitrap XL) mass spectrometer (Thermo Fisher). The mass spectrometer was operated in the data-dependent mode to automatically switch between Orbitrap-MS and LTQ-MS/MS acquisition. Survey full scan MS spectra (from m/z 300 to 1500) were acquired in the Orbitrap resolution R = 60,000 at m/z 400. Up to ten most intense ions were in parallel selected for fragmentation on the linear ion trap using collision induced dissociation at a target value of 100,000 ions and subsequently dynamically excluded for 30 s. MS/MS spectra were exported from the Progenesis software as Mascot Generic file (mgf) and used for peptide identification using Mascot (Matrix Science, London, UK; http://www.matrixscience.com), the Uniprot database (http://www.uniprot.org) restricted to mammalian entries, the Ensembl bovine database (http://www.ensembl.org) in particular, and the EMBL-Bank (http://www.ebi.ac.uk/embl/) for nucleotide sequences. A protein was considered as identified, if the confidence score was higher than 30 at a significance threshold for the Mascot result of p ≤ 0.01.

### Gene ontology project term classification of MDBK cell surface proteins

Proteins identified on MDBK cell surface by LC-MS/MS were used for annotation of GO terms “cellular component”. GO terms were retrieved from the Ensembl database and AmiGO (AmiGO version 1.8; GO database release 2012-12-08; http://amigo.geneontology.org), either directly from the entries in the bovine database or by searching orthologes in human, mouse or rat.

## Abbreviations

AK: Adenylate kinase; BNP: Bovine neonatal pancytopenia; BVD(V): Bovine Viral Diarrhoea (virus); CD41: Integrin alpha-IIb; GAPDH: Glyceraldehyde-3-phosphate dehydrogenase; GO terms: Gene ontology project terms; Hsc70: Heat shock cognate 70 kDa protein 8; LC-MS/MS: Liquid-chromatography mass spectrometry/mass spectrometry; MHC I: Major histocompatibility complex class I; MDBK: Madin-darby bovine kidney; (m)M: (milli)Molar; min: Minutes; SDS-PAGE: Sodium dodecyl sulfate polyacrylamide gel electrophoresis; TGF-ß: Transforming growth factor ß; TRALI: Transfusion-related acute lung injury; TSP 1: Thrombospondin-1; VDBP: Vitamin D-binding protein.

## Competing interests

No conflicts of interest, financial or otherwise, are declared by the authors.

## Authors’ contributions

KN performed cell surface labelling, analyzed the results and wrote the manuscript. SMH carried out mass spectrometry analysis, wrote parts of the manuscript and revised the article. MU contributed to the conception and revision of the article. CAD designed the study, evaluated the results and drafted the article. All authors read and approved the final manuscript.

## Supplementary Material

Additional file 1: **Summary of proteins identified in vaccine A, vaccine B and on MDBK cell surface In total, 310 proteins were identified in vaccine A, vaccine B and on MDBK cell surface.** A: Number of protein in list order. B: Protein name. C: Accession number as listed on Ensembl database (http://www.ensembl.org) or in EMBL Database (http://www.ebi.ac.uk/embl/), respectively. D: Molecular weight in kDa. *x* indicates that the protein was identified in the according preparation. Proteins listed were identified by LC-MS/MS with a probability score that is significant with p < 0.05 if the confidence score was >30 at a significance threshold for the Mascot result of p ≤ 0.01. (DOC 347 kb) Click here for file
