# Supplementary material for: Bovine neonatal pancytopenia - Comparative proteomic characterization of two BVD vaccines and the producer cell surface proteome (MDBK)
Source: BMC Vet Res. 2013 Jan 23;9:18. doi: 10.1186/1746-6148-9-18 (PMC3560244; doi:10.1186/1746-6148-9-18)
Supplement: Additional file 1: — Summary of proteins identified in vaccine A, vaccine B and on MDBK cell surface In total, 310 proteins were identified in vaccine A, vaccine B and on MDBK cell surface. A: Number of protein in list order. B: Protein name. C: Accession number as listed on Ensembl database (http://www.ensembl.org) or in EMBL Database (http://www.ebi.ac.uk/embl/), respectively. D: Molecular weight in kDa. x indicates that the protein was identified in the according preparation. Proteins listed were identified by LC-MS/MS with a probability score that is significant with p < 0.05 if the confidence score was >30 at a significance threshold for the Mascot result of p ≤ 0.01. (DOC 347 kb) [file 1746-6148-9-18-S1.doc]

### Summary of proteins identified in vaccine A, vaccine B and on MDBK cell surface.

| **No.** | **Identified Protein** | **Accession Number** | **MW (kDa)** | **Vac.B** | **Vac.A** | **MD**  **BK** |
| --- | --- | --- | --- | --- | --- | --- |
| 1 | Alpha-2-macroglobulin | ENSBTAP00000006167 | 168 | x | x | x |
| 2 | Annexin A1 | ENSBTAP00000021256 | 39 | x | x | x |
| 3 | Keratin, type I cytoskeletal 10 | ENSBTAP00000017140 | 59 | x | x | x |
| 4 | Myosin, heavy chain 9, non-muscle V | ENSBTAP00000013737 | 227 | x | x | x |
| 5 | Serotransferrin | ENSBTAP00000009564 | 78 | x | x | x |
| 6 | Serpin A3-1 | ENSBTAP00000042480 | 46 | x | x | x |
| 7 | Serum albumin | ENSBTAP00000022763 | 69 | x | x | x |
| 8 | Vimentin | ENSBTAP00000024572 | 54 | x | x | x |
| 9 | 40S ribosomal protein S3 | ENSBTAP00000003962 | 27 |  | x | x |
| 10 | 40S ribosomal protein S4 | ENSBTAP00000052226 | 30 |  | x | x |
| 11 | 60S ribosomal protein L7a | ENSBTAP00000015358 | 30 |  | x | x |
| 12 | Actin, cytoplasmic 2, N-terminally processed | ENSBTAP00000008132 | 42 |  | x | x |
| 13 | Adenylate kinase 2, mitochondrial | ENSBTAP00000023406 | 26 |  | x | x |
| 14 | Alpha-1-acid glycoprotein | ENSBTAP00000022991 | 23 |  | x | x |
| 15 | Alpha-1-antiproteinase | ENSBTAP00000004927 | 46 |  | x | x |
| 16 | Alpha-2-HS-glycoprotein | ENSBTAP00000000673 | 38 |  | x | x |
| 17 | Alpha-actinin-4 | ENSBTAP00000014894 | 105 |  | x | x |
| 18 | Alpha-enolase | ENSBTAP00000017839 | 47 |  | x | x |
| 19 | ATP synthase subunit alpha, mitochondrial | ENSBTAP00000010806 | 60 |  | x | x |
| 20 | ATP synthase subunit beta, mitochondrial | ENSBTAP00000017710 | 56 |  | x | x |
| 21 | Complement C3 | ENSBTAP00000022979 | 187 |  | x | x |
| 22 | Complement C4 | ENSBTAP00000009019 | 102 |  | x | x |
| 23 | Endopin 2 | ENSBTAP00000011576 | 47 |  | x | x |
| 24 | Glyceraldehyde-3-phosphate dehydrogenase | ENSBTAP00000037577 | 36 |  | x | x |
| 25 | Heat shock cognate 70 kDa protein 8 | ENSBTAP00000017497 | 71 |  | x | x |
| 26 | Hemopexin | ENSBTAP00000004635 | 52 |  | x | x |
| 27 | Histone H2B type 1 | ENSBTAP00000024155 | 25 |  | x | x |
| 28 | keratin, type II cytoskeletal 8 | ENSBTAP00000001108 | 54 |  | x | x |
| 29 | MHC class I heavy chain isoform 1 | ENSBTAP00000031126 | 40 |  | x | x |
| 30 | Pyruvate kinase | ENSBTAP00000044619 | 61 |  | x | x |
| 31 | Sodium/potassium-transporting ATPase subunit alpha-1 | ENSBTAP00000001646 | 113 |  | x | x |
| 32 | Thrombospondin-1 | ENSBTAP00000002600 | 129 |  | x | x |
| 33 | Vitamin D-binding protein | ENSBTAP00000033386 | 53 |  | x | x |
| 34 | Alpha-S1-casein | ENSBTAP00000010119 | 25 | x |  | x |
| 35 | Kappa-casein | ENSBTAP00000028685 | 21 | x |  | x |
| 36 | Keratin, type II cytoskeletal 5 | ENSBTAP00000009478 | 62 | x |  | x |
| 37 | 14-3-3 protein gamma, N-terminally processed | ENSBTAP00000005327 | 28 | x | x |  |
| 38 | Anastellin | ENSBTAP00000010925 | 272 | x | x |  |
| 39 | Annexin 5 | ENSBTAP00000028988 | 36 | x | x |  |
| 40 | Annexin A2 | ENSBTAP00000012655 | 39 | x | x |  |
| 41 | Calreticulin | ENSBTAP00000020111 | 48 | x | x |  |
| 42 | Proactivator polypeptide | ENSBTAP00000044260 / ENSBTAP00000028655 | 58 | x | x |  |
| 43 | Proteasome subunit beta type-3 | ENSBTAP00000004984 | 23 | x | x |  |
| 44 | Protein disulfide-isomerase | ENSBTAP00000007943 | 57 | x | x |  |
| 45 | Purine nucleoside phosphorylase | ENSBTAP00000016346 | 32 | x | x |  |
| 46 | Superoxide dismutase | ENSBTAP00000018580 | 26 | x | x |  |
| 47 | Triosephosphate isomerase | ENSBTAP00000026358 | 27 | x | x |  |
| 48 | Biglycan | ENSBTAP00000044462 | 42 | x |  |  |
| 49 | Calumenin | ENSBTAP00000021909 | 37 | x |  |  |
| 50 | Collagen alpha-1(III) chain | ENSBTAP00000028617 | 138 | x |  |  |
| 51 | Collagen alpha-2(I) chain | ENSBTAP00000033771 | 129 | x |  |  |
| 52 | Decorin | ENSBTAP00000004562 | 40 | x |  |  |
| 53 | Dickkopf-related protein 3 | ENSBTAP00000005599 | 38 | x |  |  |
| 54 | Dipeptidyl peptidase 3 | ENSBTAP00000026834 | 81 | x |  |  |
| 55 | Dipeptidyl peptidase 7 | ENSBTAP00000055373 | 54 | x |  |  |
| 56 | Fibromodulin | ENSBTAP00000019854 | 43 | x |  |  |
| 57 | Fibulin-2 | ENSBTAP00000005240 | 129 | x |  |  |
| 58 | Galectin-3-binding protein | ENSBTAP00000001802 | 62 | x |  |  |
| 59 | Glypican 1 | ENSBTAP00000018329 | 61 | x |  |  |
| 60 | Lumican | ENSBTAP00000002279 | 39 | x |  |  |
| 61 | Lysosome-associated membrane glycoprotein 1 | ENSBTAP00000042536 | 44 | x |  |  |
| 62 | Pigment epithelium-derived factor | ENSBTAP00000012804 | 46 | x |  |  |
| 63 | Polyprotein (Bovine viral diarrhea virus 1) | EMBL AAC61755.1 | 438 | x |  |  |
| 64 | Proteasome subunit beta type-4 | ENSBTAP00000028364 | 29 | x |  |  |
| 65 | RCN1 protein | ENSBTAP00000056127 | 39 | x |  |  |
| 66 | Secreted protein acidic and rich in cysteine | ENSBTAP00000019758 | 35 | x |  |  |
| 67 | Versican core protein | ENSBTAP00000019848 | 370 | x |  |  |
| 68 | xaa-Pro dipeptidase | ENSBTAP00000042663 | 55 | x |  |  |
| 69 | 10 kDa heat shock protein, mitochondrial | ENSBTAP00000016712 | 11 |  | x |  |
| 70 | 14-3-3 protein epsilon | ENSBTAP00000007442 | 29 |  | x |  |
| 71 | 26S proteasome non-ATPase regulatory subunit 7 | ENSBTAP00000024515 | 37 |  | x |  |
| 72 | 40S ribosomal protein S15a | ENSBTAP00000047540 | 15 |  | x |  |
| 73 | 60 kDa heat shock protein, mitochondrial | ENSBTAP00000016708 | 61 |  | x |  |
| 74 | 60S ribosomal protein L14 | ENSBTAP00000002642 | 23 |  | x |  |
| 75 | 60S ribosomal protein L5 | ENSBTAP00000002626 | 34 |  | x |  |
| 76 | 6-phosphogluconate dehydrogenase, decarboxylating | ENSBTAP00000017988 | 53 |  | x |  |
| 77 | Abhydrolase domain-containing protein 14B | ENSBTAP00000024782 | 22 |  | x |  |
| 78 | Adenylyl cyclase-associated protein 1 | ENSBTAP00000017775 | 51 |  | x |  |
| 79 | ADP/ATP translocase 3 | ENSBTAP00000055370 | 33 |  | x |  |
| 80 | Aldose reductase | ENSBTAP00000013082 | 36 |  | x |  |
| 81 | Alpha-actinin 1 | ENSBTAP00000024301 | 103 |  | x |  |
| 82 | Alpha-crystallin B chain | ENSBTAP00000000556 | 20 |  | x |  |
| 83 | Alpha-N-acetylgalactosaminidase | ENSBTAP00000012508 | 47 |  | x |  |
| 84 | Annexin A8 | ENSBTAP00000043804 | 37 |  | x |  |
| 85 | Antithrombin-III | ENSBTAP00000005713 | 52 |  | x |  |
| 86 | Apolipoprotein A-I | ENSBTAP00000002914 | 30 |  | x |  |
| 87 | Aspartate aminotransferase, mitochondrial | ENSBTAP00000009440 | 48 |  | x |  |
| 88 | Beta-2-microglobulin | ENSBTAP00000016359 | 14 |  | x |  |
| 89 | Cadherin-17 | ENSBTAP00000043215 | 93 |  | x |  |
| 90 | Carbonic anhydrase 2 | ENSBTAP00000023581 | 29 |  | x |  |
| 91 | Cathepsin L1 light chain | ENSBTAP00000000962 | 37 |  | x |  |
| 92 | Chloride intracellular channel protein 1 | ENSBTAP00000017995 | 27 |  | x |  |
| 93 | Clathrin heavy chain 1 | ENSBTAP00000022210 | 192 |  | x |  |
| 94 | Clusterin alpha chain | ENSBTAP00000007324 | 51 |  | x |  |
| 95 | Coatomer subunit gamma | ENSBTAP00000017588 | 97 |  | x |  |
| 96 | Cofilin-1 | ENSBTAP00000028602 | 19 |  | x |  |
| 97 | Collagen alpha-1(XI) chain | ENSBTAP00000028276 | 182 |  | x |  |
| 98 | Cystatin-C | ENSBTAP00000000790 | 16 |  | x |  |
| 99 | Cytosolic acyl coenzyme A thioester hydrolase | ENSBTAP00000028254 | 37 |  | x |  |
| 100 | D-3-phosphoglycerate dehydrogenase | ENSBTAP00000008907 | 56 |  | x |  |
| 101 | D-dopachrome decarboxylase | ENSBTAP00000029458 | 13 |  | x |  |
| 102 | Destrin | ENSBTAP00000020512 | 19 |  | x |  |
| 103 | EF-hand domain-containing protein D2 | ENSBTAP00000042244 | 27 |  | x |  |
| 104 | Elongation factor 1-gamma | ENSBTAP00000019318 | 50 |  | x |  |
| 105 | Elongation factor 2 | ENSBTAP00000005581 | 95 |  | x |  |
| 106 | Endoplasmin | ENSBTAP00000004364 | 92 |  | x |  |
| 107 | Epididymal secretory protein E1 | ENSBTAP00000029271 | 17 |  | x |  |
| 108 | Eukaryotic initiation factor 4A-I | ENSBTAP00000000144 | 46 |  | x |  |
| 109 | Ezrin | ENSBTAP00000013663 | 69 |  | x |  |
| 110 | Farnesyl pyrophosphate synthase | ENSBTAP00000014472 | 41 |  | x |  |
| 111 | Fatty acid synthase | ENSBTAP00000021260 | 273 |  | x |  |
| 112 | Fatty acid-binding protein, heart | ENSBTAP00000022375 | 15 |  | x |  |
| 113 | Fructose-bisphosphate aldolase C-A | ENSBTAP00000017177 | 39 |  | x |  |
| 114 | Galectin-3 | ENSBTAP00000041298 | 28 |  | x |  |
| 115 | GANAB protein | ENSBTAP00000022558 | 109 |  | x |  |
| 116 | Glucose-6-phosphate isomerase | ENSBTAP00000008386 | 65 |  | x |  |
| 117 | Glutathione S-transferase P | ENSBTAP00000004615 | 24 |  | x |  |
| 118 | Glycyl-tRNA synthetase | ENSBTAP00000025254 | 83 |  | x |  |
| 119 | GTP-binding nuclear protein Ran | ENSBTAP00000055044 | 24 |  | x |  |
| 120 | Heat shock 70kDa protein 5 | ENSBTAP00000052422 | 72 |  | x |  |
| 121 | Heat shock protein beta-1 | ENSBTAP00000015883 | 23 |  | x |  |
| 122 | Heat shock protein HSP 90-beta | ENSBTAP00000001034 | 83 |  | x |  |
| 123 | Heterogeneous nuclear ribonucleoprotein A1 | ENSBTAP00000002033 | 34 |  | x |  |
| 124 | Heterogeneous nuclear ribonucleoprotein D0 | ENSBTAP00000018540 | 38 |  | x |  |
| 125 | Heterogeneous nuclear ribonucleoprotein H2 | ENSBTAP00000009864 | 49 |  | x |  |
| 126 | Heterogeneous nuclear ribonucleoprotein K | ENSBTAP00000028162 | 51 |  | x |  |
| 127 | Heterogeneous nuclear ribonucleoproteins A2/B1 | ENSBTAP00000007527 | 36 |  | x |  |
| 128 | Hippocampal cholinergic neurostimulating peptide | ENSBTAP00000024107 | 21 |  | x |  |
| 129 | IgM heavy chain constant region | ENSBTAP00000030521 | 53 |  | x |  |
| 130 | Immunoglobulin kappa constant | ENSBTAP00000042747 | 26 |  | x |  |
| 131 | Immunoglobulin lambda-like polypeptide 1 | ENSBTAP00000041617 | 25 |  | x |  |
| 132 | Insulin-like growth factor-binding protein 2 | ENSBTAP00000007349 | 34 |  | x |  |
| 133 | Lamin A/C | ENSBTAP00000023373 | 65 |  | x |  |
| 134 | L-lactate dehydrogenase A chain | ENSBTAP00000011447 | 37 |  | x |  |
| 135 | Macrophage-capping protein | ENSBTAP00000007150 | 39 |  | x |  |
| 136 | Malate dehydrogenase, cytoplasmic | ENSBTAP00000025691 | 36 |  | x |  |
| 137 | Mesencephalic astrocyte-derived neurotrophic factor | ENSBTAP00000042500 | 20 |  | x |  |
| 138 | Nucleoside diphosphate kinase B | ENSBTAP00000041066 | 17 |  | x |  |
| 139 | Ornithine aminotransferase, mitochondrial | ENSBTAP00000009097 | 48 |  | x |  |
| 140 | PDIA6 protein | ENSBTAP00000002508 | 50 |  | x |  |
| 141 | Peptidyl-prolyl cis-trans isomerase A | ENSBTAP00000015924 | 18 |  | x |  |
| 142 | Peptidyl-prolyl cis-trans isomerase B | ENSBTAP00000022378 | 24 |  | x |  |
| 143 | Peroxiredoxin-2 | ENSBTAP00000015996 | 22 |  | x |  |
| 144 | Peroxiredoxin-5, mitochondrial | ENSBTAP00000011403 | 23 |  | x |  |
| 145 | Phosphoglycerate kinase 1 | ENSBTAP00000001187 | 45 |  | x |  |
| 146 | Phosphoglycerate mutase 1 | ENSBTAP00000032864 | 29 |  | x |  |
| 147 | Polyprotein (Bovine viral diarrhea virus 1) | EMBL ABP57735.1 | 439 |  | x |  |
| 148 | Polypyrimidine tract-binding protein 1 | ENSBTAP00000054832 | 47 |  | x |  |
| 149 | Profilin-1 | ENSBTAP00000006465 | 15 |  | x |  |
| 150 | Prohibitin | ENSBTAP00000022759 | 30 |  | x |  |
| 151 | Proteasome subunit alpha type-6 | ENSBTAP00000012773 | 25 |  | x |  |
| 152 | Proteasome subunit alpha type-7 | ENSBTAP00000006760 | 28 |  | x |  |
| 153 | Protein disulfide-isomerase A3 | ENSBTAP00000022854 | 57 |  | x |  |
| 154 | Protein HP-20 homolog | ENSBTAP00000037834 | 21 |  | x |  |
| 155 | Protein HP-25 homolog 1 | ENSBTAP00000040954 | 23 |  | x |  |
| 156 | Protein S100-A10 | ENSBTAP00000020150 | 11 |  | x |  |
| 157 | Protein S100-A11 | ENSBTAP00000020148 | 11 |  | x |  |
| 158 | Rab GDP dissociation inhibitor beta | ENSBTAP00000006992 | 50 |  | x |  |
| 159 | Ras-related protein Rab-7a | ENSBTAP00000013451 | 24 |  | x |  |
| 160 | Retinal dehydrogenase 1 | ENSBTAP00000010661 | 55 |  | x |  |
| 161 | Rho GDP-dissociation inhibitor 1 | ENSBTAP00000040280 | 23 |  | x |  |
| 162 | Ribonucleoside-diphosphate reductase M1 chain | ENSBTAP00000017428 | 90 |  | x |  |
| 163 | Ribosomal protein L22 | ENSBTAP00000000065 | 13 |  | x |  |
| 164 | RNA binding motif (RNP1, RRM) protein 3 | ENSBTAP00000024034 | 17 |  | x |  |
| 165 | Serine hydroxymethyltransferase, mitochondrial | ENSBTAP00000038059 | 56 |  | x |  |
| 166 | Serpin peptidase inhibitor, clade A, member 3 | ENSBTAP00000009264 | 46 |  | x |  |
| 167 | Serpin peptidase inhibitor, clade B (ovalbumin), member 1 | ENSBTAP00000015889 | 42 |  | x |  |
| 168 | Serum amyloid P-component | ENSBTAP00000026133 | 25 |  | x |  |
| 169 | Spliceosome RNA helicase DDX39B | ENSBTAP00000029090 | 49 |  | x |  |
| 170 | Stefin-C | ENSBTAP00000024208 | 12 |  | x |  |
| 171 | Stress-70 protein, mitochondrial | ENSBTAP00000015172 | 74 |  | x |  |
| 172 | Sulfotransferase 1A1 | ENSBTAP00000011388 | 34 |  | x |  |
| 173 | T-complex protein 1 subunit delta | ENSBTAP00000042274 | 58 |  | x |  |
| 174 | T-complex protein 1 subunit gamma | ENSBTAP00000008358 | 56 |  | x |  |
| 175 | Thioredoxin | ENSBTAP00000041860 | 12 |  | x |  |
| 176 | Transgelin-2 | ENSBTAP00000002674 | 22 |  | x |  |
| 177 | Transitional endoplasmic reticulum ATPase | ENSBTAP00000019970 | 89 |  | x |  |
| 178 | Transketolase | ENSBTAP00000053095 | 65 |  | x |  |
| 179 | Translationally-controlled tumor protein | ENSBTAP00000013402 | 20 |  | x |  |
| 180 | Transthyretin | ENSBTAP00000014585 | 16 |  | x |  |
| 181 | Tubulin alpha-3 chain | ENSBTAP00000041398 | 50 |  | x |  |
| 182 | Voltage-dependent anion-selective channel protein 1 | ENSBTAP00000017430 | 31 |  | x |  |
| 183 | WD repeat-containing protein 1 | ENSBTAP00000018092 | 66 |  | x |  |
| 184 | 40S ribosomal protein S18 | ENSBTAP00000003431 | 18 |  |  | x |
| 185 | 40S ribosomal protein S25 | ENSBTAP00000039829 | 14 |  |  | x |
| 186 | 40S ribosomal protein S9 | ENSBTAP00000008502 | 23 |  |  | x |
| 187 | 5'-nucleotidase | ENSBTAP00000054917 | 36 |  |  | x |
| 188 | 60S ribosomal protein L10 | ENSBTAP00000009803 | 25 |  |  | x |
| 189 | 60S ribosomal protein L15 | ENSBTAP00000044213 | 24 |  |  | x |
| 190 | 60S ribosomal protein L18 | ENSBTAP00000020452 | 22 |  |  | x |
| 191 | 60S ribosomal protein L23 | ENSBTAP00000004190 | 15 |  |  | x |
| 192 | 60S ribosomal protein L32 | ENSBTAP00000020323 | 16 |  |  | x |
| 193 | 60S ribosomal protein L6 | ENSBTAP00000042411 | 33 |  |  | x |
| 194 | Acetyl-CoA carboxylase 1 | ENSBTAP00000023364 | 265 |  |  | x |
| 195 | Actin, alpha skeletal muscle | ENSBTAP00000006534 | 42 |  |  | x |
| 196 | AHNAK nucleoprotein | ENSBTAP00000048477 | 587 |  |  | x |
| 197 | Alpha-1B-glycoprotein | ENSBTAP00000012837 | 54 |  |  | x |
| 198 | Alpha-S2-casein | ENSBTAP00000006590 | 26 |  |  | x |
| 199 | Apolipoprotein C-III | ENSBTAP00000016453 | 11 |  |  | x |
| 200 | Bardet-Biedl syndrome 12 protein | ENSBTAP00000009953 | 78 |  |  | x |
| 201 | Basigin | ENSBTAP00000039862 | 30 |  |  | x |
| 202 | Beta-casein | ENSBTAP00000003409 | 25 |  |  | x |
| 203 | BTB (POZ) domain containing 18 | ENSBTAP00000054938 | 73 |  |  | x |
| 204 | Calcium channel, voltage-dependent, alpha 2/delta subunit 1 | ENSBTAP00000027409 | 106 |  |  | x |
| 205 | Cathelicidin-1 | ENSBTAT00000026750 | 17 |  |  | x |
| 206 | CD166 antigen | ENSBTAP00000000097 | 65 |  |  | x |
| 207 | CD276 molecule | ENSBTAP00000026300 | 56 |  |  | x |
| 208 | CD44 antigen | ENSBTAP00000015381 | 40 |  |  | x |
| 209 | CD48 antigen | ENSBTAP00000014924 | 28 |  |  | x |
| 210 | CD58 protein | ENSBTAP00000023836 | 29 |  |  | x |
| 211 | CD59 molecule, complement regulatory protein | ENSBTAP00000002967 | 14 |  |  | x |
| 212 | CD63 antigen | ENSBTAT00000015829 | 26 |  |  | x |
| 213 | CD99 molecule | ENSBTAP00000010674 | 19 |  |  | x |
| 214 | Cell adhesion molecule 1 | ENSBTAP00000036781 | 46 |  |  | x |
| 215 | ClpX caseinolytic peptidase X homolog (E. coli) | ENSBTAP00000011038 | 67 |  |  | x |
| 216 | Coagulation factor XIII A chain | ENSBTAP00000009559 | 23 |  |  | x |
| 217 | Corneodesmosin | ENSBTAP00000028955 | 53 |  |  | x |
| 218 | Cytochrome b-245 heavy chain | ENSBTAP00000026580 | 66 |  |  | x |
| 219 | Desmoplakin | ENSBTAP00000020103 | 332 |  |  | x |
| 220 | Dystroglycan | ENSBTAP00000015385 | 97 |  |  | x |
| 221 | Epithelial membrane protein 3 | ENSBTAP00000023976 | 18 |  |  | x |
| 222 | Fibrinogen alpha chain | ENSBTAP00000002145 | 67 |  |  | x |
| 223 | Fibrinogen beta chain | ENSBTAP00000029826 | 56 |  |  | x |
| 224 | Fibrinogen gamma-B chain | ENSBTAP00000046432 | 49 |  |  | x |
| 225 | Glycoprotein Ib (platelet), alpha polypeptide | ENSBTAP00000006460 | 71 |  |  | x |
| 226 | Glycoprotein V (platelet) | ENSBTAP00000017571 | 64 |  |  | x |
| 227 | Hemoglobin subunit alpha | ENSBTAP00000022034 | 15 |  |  | x |
| 228 | Hemoglobin subunit beta | ENSBTAP00000043063 | 16 |  |  | x |
| 229 | Heterogeneous nuclear ribonucleoprotein U | ENSBTAP00000048368 | 78 |  |  | x |
| 230 | Histone H1.1 | ENSBTAP00000015499 | 10 |  |  | x |
| 231 | Histone H1x | ENSBTAP00000049910 | 22 |  |  | x |
| 232 | Histone H2A | ENSBTAP00000042479 | 6 |  |  | x |
| 233 | Histone H2B | ENSBTAP00000048179 | 13 |  |  | x |
| 234 | Histone H3.3 | ENSBTAP00000034086 | 15 |  |  | x |
| 235 | Histone H4 | ENSBTAP00000025810 | 11 |  |  | x |
| 236 | Immediate early response 3-interacting protein 1 | ENSBTAP00000044053 | 9 |  |  | x |
| 237 | Immunoglobulin heavy constant mu | ENSBTAP00000001613 | 50 |  |  | x |
| 238 | Integrin alpha M | ENSBTAP00000054571 | 128 |  |  | x |
| 239 | Integrin alpha-3 | ENSBTAP00000025974 | 116 |  |  | x |
| 240 | Integrin alpha-6 | ENSBTAP00000022960 | 119 |  |  | x |
| 241 | Integrin alpha-IIb | ENSBTAP00000010741 | 114 |  |  | x |
| 242 | Integrin alpha-L | ENSBTAP00000042497 | 63 |  |  | x |
| 243 | Integrin alpha-V | ENSBTAP00000026553 | 116 |  |  | x |
| 244 | Integrin beta | ENSBTAP00000013173 | 87 |  |  | x |
| 245 | Integrin beta-1 | ENSBTAP00000021156 | 88 |  |  | x |
| 246 | Integrin beta-2 | ENSBTAP00000022687 | 84 |  |  | x |
| 247 | Intercellular adhesion molecule 3 | ENSBTAP00000020903 | 60 |  |  | x |
| 248 | Junction plakoglobin | ENSBTAP00000023522 | 82 |  |  | x |
| 249 | Junctional adhesion molecule A | ENSBTAP00000023725 | 32 |  |  | x |
| 250 | Keratin 1 | ENSBTAP00000055471 | 63 |  |  | x |
| 251 | Keratin 16 | ENSBTAP00000045066 | 40 |  |  | x |
| 252 | Keratin 2 | ENSBTAP00000054865 | 64 |  |  | x |
| 253 | Keratin 3 | ENSBTAP00000054280 | 63 |  |  | x |
| 254 | Keratin, type I cuticular Ha4 | ENSBTAP00000012807 | 45 |  |  | x |
| 255 | Keratin, type I cytoskeletal 14 | ENSBTAP00000036252 | 52 |  |  | x |
| 256 | Keratin, type I cytoskeletal 17 | ENSBTAP00000008948 | 49 |  |  | x |
| 257 | Keratin, type I cytoskeletal 25 | ENSBTAP00000040707 | 49 |  |  | x |
| 258 | Keratin, type II cuticular Hb3 | ENSBTAP00000008857 | 54 |  |  | x |
| 259 | Keratin, type II cuticular Hb5 | ENSBTAP00000000515 | 56 |  |  | x |
| 260 | Keratin, type II cytoskeletal 4 | ENSBTAP00000016832 | 58 |  |  | x |
| 261 | Keratin, type II cytoskeletal 59 kDa, component IV | ENSBTAP00000044069 | 61 |  |  | x |
| 262 | Keratin, type II cytoskeletal 7 | ENSBTAP00000021516 | 52 |  |  | x |
| 263 | Keratin, type II cytoskeletal 80 | ENSBTAP00000007288 | 88 |  |  | x |
| 264 | Kininogen-1 isoform I | ENSBTAP00000006755 | 48 |  |  | x |
| 265 | L1 cell adhesion molecule | ENSBTAP00000017906 | 140 |  |  | x |
| 266 | Lactoferricin-B | ENSBTAP00000001704 | 78 |  |  | x |
| 267 | Leukocyte antigen CD37 | ENSBTAP00000015173 | 32 |  |  | x |
| 268 | Leukocyte surface antigen CD47 | ENSBTAP00000048646 | 33 |  |  | x |
| 269 | Leukocyte surface antigen CD53 | ENSBTAP00000008479 | 24 |  |  | x |
| 270 | Leukosialin | ENSBTAP00000037201 | 50 |  |  | x |
| 271 | Low-density lipoprotein receptor | ENSBTAP00000016342 | 93 |  |  | x |
| 272 | L-selectin | ENSBTAP00000044113 | 42 |  |  | x |
| 273 | Malate dehydrogenase, mitochondrial | ENSBTAP00000012454 | 30 |  |  | x |
| 274 | Mannose receptor, C type 2 | ENSBTAP00000020897 | 163 |  |  | x |
| 275 | Membrane cofactor protein | ENSBTAP00000007107 | 40 |  |  | x |
| 276 | MYB binding protein (P160) 1a | ENSBTAP00000009771 | 152 |  |  | x |
| 277 | Myelin protein zero-like protein 1 | ENSBTAP00000003653 | 29 |  |  | x |
| 278 | Myeloperoxidase | ENSBTAP00000016989 | 82 |  |  | x |
| 279 | MYH7 protein | ENSBTAP00000053217 | 26 |  |  | x |
| 280 | Myosin-Id | ENSBTAP00000020634 | 116 |  |  | x |
| 281 | Neuroplastin | ENSBTAP00000010806 | 31 |  |  | x |
| 282 | Neutrophil gelatinase-associated lipocalin isoform 2 | ENSBTAP00000026146 | 23 |  |  | x |
| 283 | Non-classical MHC class I antigen | ENSBTAP00000021451 | 39 |  |  | x |
| 284 | Nucleolar protein 56 | ENSBTAP00000025042 | 66 |  |  | x |
| 285 | Phosphoglycerate mutase 2 | ENSBTAP00000019336 | 29 |  |  | x |
| 286 | Platelet factor 4 | ENSBTAP00000015874 | 13 |  |  | x |
| 287 | Plexin B1 | ENSBTAP00000017840 | 232 |  |  | x |
| 288 | Plexin B2 | ENSBTAP00000019923 | 205 |  |  | x |
| 289 | Podocalyxin-like | ENSBTAP00000013799 | 54 |  |  | x |
| 290 | Propionyl-CoA carboxylase alpha chain, mitochondrial | ENSBTAP00000014120 | 81 |  |  | x |
| 291 | Prostaglandin F2 receptor negative regulator | ENSBTAP00000048973 | 99 |  |  | x |
| 292 | Protein tyrosine phosphatase, receptor type, C | ENSBTAP00000013320 | 143 |  |  | x |
| 293 | Proteoglycan 3 | ENSBTAP00000002601 | 25 |  |  | x |
| 294 | Pyruvate carboxylase, mitochondrial | ENSBTAP00000026258 | 130 |  |  | x |
| 295 | Ribosomal protein S6 kinase alpha-1 | ENSBTAP00000037744 | 83 |  |  | x |
| 296 | RNA binding motif protein 39 | ENSBTAP00000002491 | 59 |  |  | x |
| 297 | Sodium/potassium-transporting ATPase subunit beta-1 | ENSBTAP00000044161 | 15 |  |  | x |
| 298 | Sodium/potassium-transporting ATPase subunit beta-3 | ENSBTAP00000018795 | 32 |  |  | x |
| 299 | Sodium-dependent phosphate transport protein 2B | ENSBTAP00000002023 | 76 |  |  | x |
| 300 | Solute carrier family 12 member 2 | ENSBTAP00000012701 | 130 |  |  | x |
| 301 | Synaptonemal complex protein 2-like | ENSBTAP00000041952 | 97 |  |  | x |
| 302 | TAO kinase 2 | ENSBTAP00000023163 | 119 |  |  | x |
| 303 | T-cell surface glycoprotein CD5 | ENSBTAP00000018258 | 54 |  |  | x |
| 304 | Testicular cell adhesion molecule 1 homolog (mouse), pseudogene | ENSBTAP00000034654 | 60 |  |  | x |
| 305 | Trypstatin | ENSBTAP00000020817 | 39 |  |  | x |
| 306 | Tubulin alpha-1B chain | ENSBTAP00000016242 | 50 |  |  | x |
| 307 | Tubulin, beta 1 | ENSBTAP00000025008 | 50 |  |  | x |
| 308 | Vasorin | ENSBTAP00000055273 | 71 |  |  | x |
| 309 | Vitronectin | ENSBTAP00000021497 | 54 |  |  | x |
| 310 | Von Willebrand factor | ENSBTAP00000016273 | 308 |  |  | x |

In total, 310 proteins were identified in vaccine A, vaccine B and on MDBK cell surface. Accession number as listed on Ensembl database (http://www.ensembl.org) or in EMBL Database (http://www.ebi.ac.uk/embl/), respectively. *x* indicates that the protein was identified in the according preparation.

Proteins listed were identified by LC-MS/MS with a probability score that is significant with p < 0.05 if the confidence score was >30 at a significance threshold for the Mascot result of p ≤ 0.01.
